# Clinical, Epidemiologic, Histopathologic and Molecular Features of an Unexplained Dermopathy

**DOI:** 10.1371/journal.pone.0029908

**Published:** 2012-01-25

**Authors:** Michele L. Pearson, Joseph V. Selby, Kenneth A. Katz, Virginia Cantrell, Christopher R. Braden, Monica E. Parise, Christopher D. Paddock, Michael R. Lewin-Smith, Victor F. Kalasinsky, Felicia C. Goldstein, Allen W. Hightower, Arthur Papier, Brian Lewis, Sarita Motipara, Mark L. Eberhard, for the Unexplained Dermopathy Study Team

**Affiliations:** 1 Division of TB Elimination, Centers for Disease Control and Prevention, Atlanta, Georgia, United States of America; 2 Division of Research, Kaiser Permanente Northern California, Oakland, California, United States of America; 3 HIV, STD, and Hepatitis Branch, Health and Human Services Agency, County of San Diego, San Diego, California, United States of America; 4 Division of Food, Waterborne and Environmental Diseases, Centers for Disease Control and Prevention, Atlanta, Georgia, United States of America; 5 Division of Parasitic Diseases and Malaria, Centers for Disease Control and Prevention, Atlanta, Georgia, United States of America; 6 Division of High Consequence Pathogens and Pathology, Centers for Disease Control and Prevention, Atlanta, Georgia, United States of America; 7 Environmental Pathology, Joint Pathology Center, Silver Spring, Maryland, United States of America; 8 Office of Research & Development, United States Department of Veterans Affairs, Washington, District of Columbia, United States of America; 9 Department of Neurology, Emory University School of Medicine, Atlanta, Georgia, United States of America; 10 Department of Dermatology, University of Rochester School of Medicine, Rochester, New York, United States of America; 11 Division of Health Studies, Agency for Toxic Substances and Disease Registry, Atlanta, Georgia, United States of America; Université de Technologie de Compiègne, France

## Abstract

**Background:**

Morgellons is a poorly characterized constellation of symptoms, with the primary manifestations involving the skin. We conducted an investigation of this unexplained dermopathy to characterize the clinical and epidemiologic features and explore potential etiologies.

**Methods:**

A descriptive study was conducted among persons at least 13 years of age and enrolled in Kaiser Permanente Northern California (KPNC) during 2006–2008. A case was defined as the self-reported emergence of fibers or materials from the skin accompanied by skin lesions and/or disturbing skin sensations. We collected detailed epidemiologic data, performed clinical evaluations and geospatial analyses and analyzed materials collected from participants' skin.

**Results:**

We identified 115 case-patients. The prevalence was 3.65 (95% CI = 2.98, 4.40) cases per 100,000 enrollees. There was no clustering of cases within the 13-county KPNC catchment area (p = .113). Case-patients had a median age of 52 years (range: 17–93) and were primarily female (77%) and Caucasian (77%). Multi-system complaints were common; 70% reported chronic fatigue and 54% rated their overall health as fair or poor with mean Physical Component Scores and Mental Component Scores of 36.63 (SD = 12.9) and 35.45 (SD = 12.89), respectively. Cognitive deficits were detected in 59% of case-patients and 63% had evidence of clinically significant somatic complaints; 50% had drugs detected in hair samples and 78% reported exposure to solvents. Solar elastosis was the most common histopathologic abnormality (51% of biopsies); skin lesions were most consistent with arthropod bites or chronic excoriations. No parasites or mycobacteria were detected. Most materials collected from participants' skin were composed of cellulose, likely of cotton origin.

**Conclusions:**

This unexplained dermopathy was rare among this population of Northern California residents, but associated with significantly reduced health-related quality of life. No common underlying medical condition or infectious source was identified, similar to more commonly recognized conditions such as delusional infestation.

## Introduction

Morgellons is a lay term that has been used to describe an unexplained constellation of symptoms, with the primary manifestations involving the skin. Persons who identify themselves as having the condition typically report poorly or non-healing skin lesions, excretion/emergence of fibers or solid material from the skin, and pruritus or other disturbing cutaneous sensations such as formication, stinging and biting, or a pins-and-needles sensation. These symptoms are usually described as being chronic and recurrent [1].

Persons who suffer from this unexplained dermopathy sometimes also report various non-cutaneous symptoms such as generalized fatigue, difficulty concentrating, short-term memory loss and depressed mood. Some report co-morbid conditions such as chronic fatigue syndrome, fibromyalgia, neurocognitive deficits, neurological conditions such as multiple sclerosis, and psychiatric disorders. Although no fatalities have been proven to have resulted directly from this condition, some reports suggest that persons with the condition have experienced substantial declines in quality of life, including social disruption and isolation, decreased work productivity or job loss, and total disability [2].

This condition is not currently recognized as a distinct clinical disorder with established diagnostic criteria that are generally accepted by the medical community and many dermatologists consider the condition to be synonymous with delusional parasitosis (DP). To date, most of what is known about the condition is based on isolated case reports or anecdotal accounts. A range of potential infectious (e.g., Lyme disease, parasitic) and non-infectious causes has been postulated, but the etiology of this condition remains unknown and there have been no proven effective medical therapies.

Over the past few years, the Centers for Disease Control and Prevention (CDC) and several state and local health departments have received an increasing number of inquiries from the public and providers regarding this condition. In response, we conducted an investigation in Northern California where a possible cluster of illness was reported by local public health officials. We sought to better characterize the clinical and epidemiologic features of this condition, to estimate its prevalence in a defined population, and to generate hypotheses about causative or contributory factors. In this report, we present the findings.

## Methods

### Study Population

This study was conducted among enrollees of Kaiser Permanente of Northern California (KPNC) during July 2006 through June 2008. KPNC is an integrated, managed care consortium that has approximately 3.2 million enrollees, representing nearly 30% of the population in 13 Northern California counties. The membership is sociodemographically and culturally diverse, but highly representative of the general population [3].

### Study Design and Eligibility Criteria

This descriptive case series study had three major components: a cross-sectional survey, clinical evaluations, and histopathologic studies. To be eligible for study enrollment, a KPNC member had to be ≥13 years of age and English-speaking. Participants provided written informed consent for the clinical examination, including photographic documentation of their skin (total body and lesion images) and collection of all clinical samples, including biopsies, blood, urine and fibers/materials. The study was reviewed and approved by Institutional Review Boards at CDC, KPNC, the Armed Forces Institute of Pathology (AFIP), and the University of Rochester.

### Case definition

A case-patient was defined as any person who was a member of, and received care at, KPNC for any period between July 1, 2006 and June 30, 2008 (case-finding period) and reported fibers, threads, specks, dots, fuzzballs, granules or other forms of solid material coming out of his/her skin; AND one or both of the following:

- a skin lesion such as a rash, wound, ulcer, or nodule; OR
- a disturbing skin symptom such as pruritus, feeling that something is crawling on top of or under the skin, or stinging, biting, or a pins and needles sensation.

### Case-finding

To identify cases, we first searched the electronic health records of KNPC enrollees to identify clinic visits with certain keywords (i.e, “Morgellons”, “fiber”, “thread”, “fuzzball”, “dots”, “specks”, “granules”, “delusion”) recorded in the progress notes or with the ICD-9-CM code 300.29 (delusions, parasitosis), the code used at KPNC for patients with Morgellons. The search was limited to dermatology, psychiatry, infectious diseases, pediatric, and primary care clinic visits.

Next, study team members used pre-defined criteria to review the medical records of all persons identified by the electronic search to determine if they had suggestive signs and symptoms. Finally, persons who met medical records review criteria were screened by telephone by a member of the research team using a standardized tool to determine if they met the case definition; those who met the case definition were invited to enroll in the study.

Persons who were not captured by the electronic search but contacted KPNC research staff seeking study participation also were screened by telephone to determine if they met the case definition. If so, they were invited to enroll in the study.

### Prevalence Estimates and Geospatial Analysis

To estimate the prevalence of cases, we used as our denominator the average monthly KPNC enrollment during July 2006–June 2008. We enumerated total, and age- and sex-specific monthly enrollment. Because the electronic health record (EHR) was being introduced during the case-finding period, we used only data from facilities in which the EHR was functioning (and therefore identifying potential cases) in each monthly denominator. Case-patients who self-identified were not included in the numerators of the rate estimates. Geospatial analysis was done to evaluate the possibility of geographic clustering of cases in KPNC's 13-county catchment area.

### Cross-sectional Survey

Persons who were study eligible were invited to complete a self-administered, Internet-based survey (or alternatively a telephone administered survey) to collect detailed epidemiologic data. Information collected at the survey included additional demographic data, household information, prior medical history, details regarding skin symptoms, pet ownership, travel history, tobacco, alcohol and illicit drug use history, and potential environmental exposures in the home or community. The SF-12 v2 Health Survey® (QualityMetrics) was used to assess measures of health-related quality of life, including self-perceived health status and well-being and Physical Component Scores (PCS) and Mental Component Scores (MCS). Mean PCS and MCS were compared with expected norms of 50 (standard deviation [SD] of 10) for the US population [4].

### Clinical Evaluation

Additional inclusion criteria for the clinical examination were: age ≥18 years; self-reported active skin symptoms (consistent with the case definition) within the preceding 2 weeks, and current membership in KPNC. All components of the clinical evaluation were done at a Kaiser Permanente's Division of Research in Oakland. Standardized data collection forms were used for all components of the examination.

#### Clinical examinations

A medical history and a general physical examination were administered by an internist. A dermatologist administered a separate examination which included documentation of skin findings, collection of skin biopsies, and collection of fibers or other material present on the skin. Total body photographs were done by a medical photographer to document case-patients' overall skin condition and the distribution of lesions.

Standardized criteria were used to categorize lesions and grade (normal, mild, moderate, severe) the extent of skin abnormalities. The number, location and types of lesions were recorded by body area. Additional comments were included, as appropriate, to record clinical impressions that were not captured adequately by the standardized form. Participants self-rated the severity of their skin symptoms within the 24 hours preceding examination, using a Likert scale.

#### Collection of Skin Samples and Foreign Material

Skin samples were obtained using a 4 mm punch biopsy. Biopsies, up to five per participant, were obtained from abnormal and clinically normal skin areas. An abnormal skin area was defined as one that had either abnormal appearance (to both participant and examiner) or abnormal sensation (although appearing normal). Two biopsies were possible per abnormal skin area (one for histopathologic analysis and, if clinically indicated, one for microbiologic culture); a single biopsy was obtained from normal skin. A dermatoscope was used to photograph each biopsy site before and after the procedure. Fibers or potential foreign material present on the participant's skin were photographed, then collected and placed in a formalin-filled plastic container and sent for analysis. Only materials collected from participants' skin by the dermatologist were sent for analysis. At study completion, an independent review of all dermatologic examination reports, dermatologic photographs and pathology reports was done by a second dermatologist.

#### Neurocognitive and Neuropsychiatric Testing

Participants were administered a battery of standardized neuropsychological tests. The Wechsler Test of Adult Reading (WTAR) was used to provide an estimate of intellectual functioning. Cognitive function was assessed using the: Stroop Color and Word Test; the Proverbs and Verbal Fluency measures of the Delis-Kaplan Executive Function System™ (D-KEFS™); Ruff 2 & 7 Selective Attention Test; Brief Visuospatial Memory Test-Revised (BVMT-R™); Hopkins Verbal Learning Test-Revised™; and the Trail Making Test (TMT) Parts A & B. The PAI® (Personality Assessment Inventory™) was used to assess personality functioning and to screen for evidence of major psychiatric disorders [5]. All tests were administered in-person, scored and interpreted by trained neuropsychologists.

Raw scores on the cognitive tests and PAI scales were converted to T-scores using demographically adjusted normative data. T-scores ≤30 (corresponding to 2 Standard Deviations (SD) below the normative mean [50, SD = 10]) on the cognitive measures were considered clinically significant. T-scores >70 on the Personality Scales were considered clinically significant.

### Laboratory Studies

#### Blood samples and serologic tests

Blood was obtained for complete blood count with differential, erythrocyte sedimentation rate (ESR), serum glucose, serum ferritin, serum IgE, liver function tests, albumin, total protein, serum total calcium, phosphorus, serum blood urea nitrogen (BUN) and creatinine, Vitamin B12 and folate, serum Vitamin B1, antinuclear antibody (ANA), rheumatoid factor (RF), thyroid function tests, and C-reactive protein (CRP).

Serum samples were tested for hepatitis B surface antigen (HBsAg) and antibodies to hepatitis B core antigen (anti-HBc), hepatitis B surface antigen (anti-HBs), and hepatitis C (anti-HCV). Past or present HBV infection was defined as the presence of anti-HBc. Persons with test results positive for anti-HBs and negative for anti-HBc were considered to have vaccine-induced immunity.

At CDC, serum samples were tested for the presence of antibodies to *Borrelia burgdorferi*, *Toxocara* and *Strongyloides*. *B. burgdorferi* seroreactivity was determinedusing a polyvalent (IgM/IgG) enzyme immunoassay (EIA) (Vidas; BioMérieux Vitek, Hazelwood, MO) and separate IgM and IgG Western blot (WB) (Marblot; MarDx Diagnostics, Carlsbad, CA). Low-passage *B. burgdorferi*, strain B31, was used as the antigen source for both assays. *B. burgdorferi* seropositivity was defined based on the IgG WB as serum specimens were collected 130 days after illness onset [6]. Specimens with at least 5 of 10 IgG diagnostic bands by WB were considered positive, in accordance with the CDC-recommended criteria [6].

To detect *Toxocara* antibodies, a *Toxocara* EIA that detects both *T. canis* and *T. cati* infections was used. The assay utilizes *T. canis* excretory-secretory (TES) antigens from infective-stage larvae and antibodies are measured and reported as a titer. A positive *Toxocara* result was defined as a titer ≥1∶32 [7].

Seropositivity to *Strongyloides* was determined using a quantitative ELISA which measures antibodies to a crude larval extract purified from infective third-stage larvae of *S. stercoralis*. A positive *Strongyloides* result was defined as a value ≥1∶7 [8].

#### Non-blood samples and tests

Urine was obtained for microscopy and, if pyuria or bacteriuria was detected, sent for culture. Because of the association between drug use and formication, participants' hair samples were tested to determine the presence of amphetamines, barbiturates, benzodiazepines, cocaine, cannabinoids, methadone, opiates, phencyclidine and propoxyphene (HairConfirm, Craig Medical Distribution, Inc., Vista CA). Chest radiographs also were performed.

Swabs obtained from open skin lesions were sent to Quest Diagnostics® for microbiologic analysis, including gram stain, bacterial and fungal cultures. Viral cultures were performed if the lesions were clinically consistent with a viral etiology.

#### Histopathologic, immunohistochemical, molecular qnd chemical evaluation of biopsy specimens

Skin biopsy samples were placed in 10% formalin and sent to CDC. 3 µm sections were prepared, stained with hematoxylin and eosin (H&E) and evaluated by a team of infectious diseases pathologists for histopathologic changes, using light microscopy and for exogenous material, using polarized light. Specimens showing inflammatory cell infiltrates on light microscopy were further evaluated on additional sections using special stains (i.e., Lillie-Twort, Warthin-Starry, and Grocott methenamine silver techniques) to detect bacteria or fungi and, if an infectious agent was identified, by immunohistochemical (IHC) stains, and/or PCR assays [9]–[13].

H&E-stained slides prepared at CDC were anonymized and sent to AFIP, along with two unstained, consecutive cuts from the same biopsy (one mounted on a carbon disc, the other on an aluminum coated slide) to maximize the probability of detection and analysis of fibers or material in the sections. At AFIP, H&E-stained sections were evaluated by light microscopy by two dermatopathologists who were blinded to the clinical diagnosis of the cases and under polarized light by a pathologist with experience in the evaluation of unidentified materials in tissue sections. Tissue sections containing unidentified material were further analyzed by scanning electron microscopy with energy dispersive X-ray analysis (SEM/EDXA) to determine the material's elemental composition and by infrared spectroscopy (IR) to identify molecular characteristics [14], [15]. The measured infrared spectra were compared with those of authentic samples such as cotton gauze (for cellulose), and to spectra stored in a digital spectral library.

#### Molecular and Spectral Analysis of Fibers and Other Material

Fibers or other materials collected from participants' skin were analyzed at AFIP. Submitted materials were photographed, attached to aluminized slides by drying, crushing or by using conducting adhesive tabs (Polysciences Inc., Warrington, PA) and then analyzed by SEM/EDXA and IR [14], [15].

### Statistical analysis

Continuous data were summarized using descriptive statistics, including mean, standard deviation, minimum, maximum, median and inter quartile range. Categorical data was summarized using frequency counts and percents; confidence limits around point estimates were determined, where indicated. Categorical variables were compared using chi-square tests. Census block data were analyzed using ArcGIS Geographic Information System software and SaTScan cluster analysis software to determine geospatial patterns and assess geographic clustering of the cases by place of residence.

## Results

### Case Finding and Characteristics, Prevalence Estimates and Geospatial Mapping

**Figure 1:**
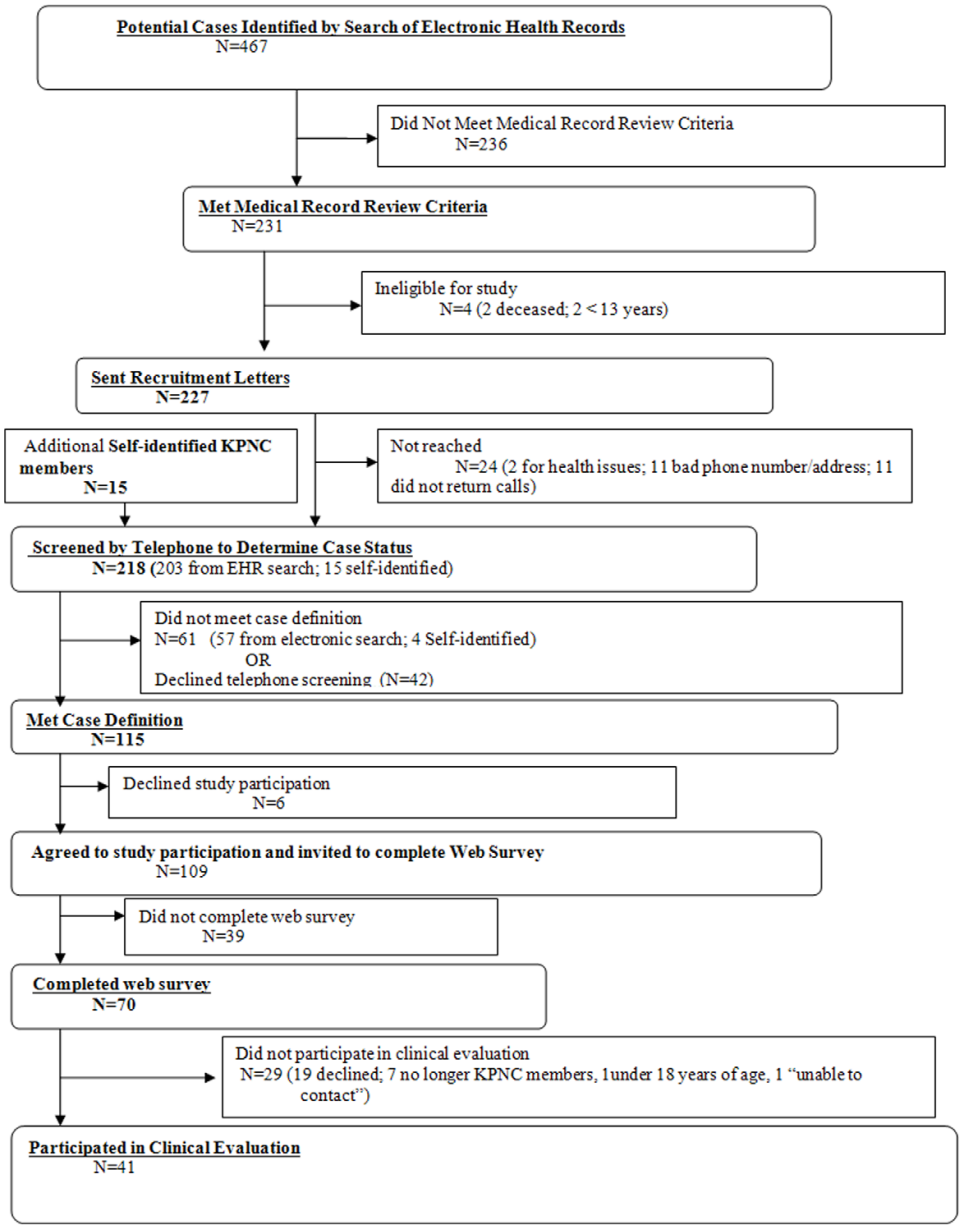
Summary of Case Finding and Study Enrollment Efforts, Unexplained Dermopathy, Calilfornia.

**Figure 2:**
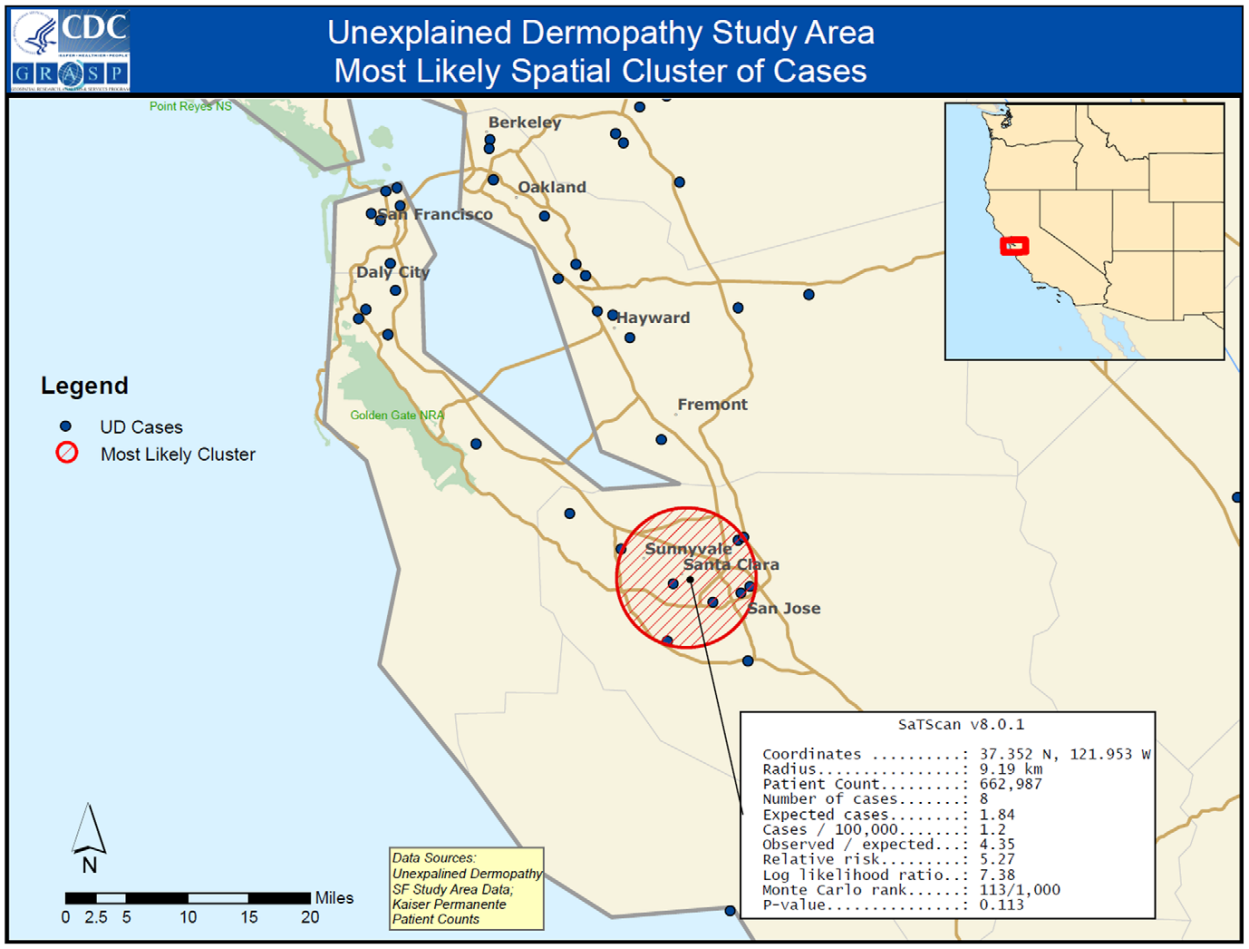
Geospatial Mapping of UD Cases By Place of Residence, California.

The summary of case-finding and study enrollment efforts are shown in Figure 1. A total of 115 KPNC enrollees met our case definition; 104 (90.4%) were identified by search of electronic health records, representing a prevalence of 3.65 (95% CI = 2.98, 4.40) per 100,000 enrollees. The rate was higher among females than males and highest amongst persons 45–64 years of age (Table 1). Eleven additional KPNC members who self-identified also met the case definition and eligibility criteria for study participation. There was no geospatial clustering of cases within the 13-county catchment area served by KPNC (p = .113) (Figure 2).

**Table 1:** Age-and Sex-specific Prevalence Rates of Unexplained Dermopathy, California, July 2006–June 2008.

	No. enrollees	No. cases**	Rate* (95% CI)
**TOTAL**	2,850,606	104	3.65 (2.98, 4.40)
**Sex**			
Female	1,469,118	79	5.38 (4.26, 6.70)
Male	1,381,488	25	1.81 (1.17, 2.67)
**Age group**			
<18	685,918	1	0.15 (0.004, 0.81)
18–44	1,007,843	21	2.08 (1.29, 3.18)
45–64	801,267	65	8.11 (6.26, 10.34)
> = 65	355,568	17	4.78 (2.79, 7.65)

*rate per 100,000 enrollees.

**excludes 11 cases not identified by electronic record.

Case-patients had a median age of 55 years (range: 17–93); 89 (77.4%) were female. When screened for study eligibility, 81/115 (70.4%) case-patients identified the material emerging from their skin as “fibers” (alone or in combination with other materials); the remaining 34 (29.6%) identified the materials as other than fibers, including specks (59%), granules (56%), dots (50%), worms (35%), sand (32%), eggs (32%), fuzzballs (21%), and larvae (15%).

### Cross-sectional Survey

A total of 70 (61%) case-patients completed the cross-sectional survey. Case-patients who completed the survey were more likely to be female (p = .04), but did not differ by age when compared with case-patients who did not complete the survey (data not shown).

**Figure 3:**
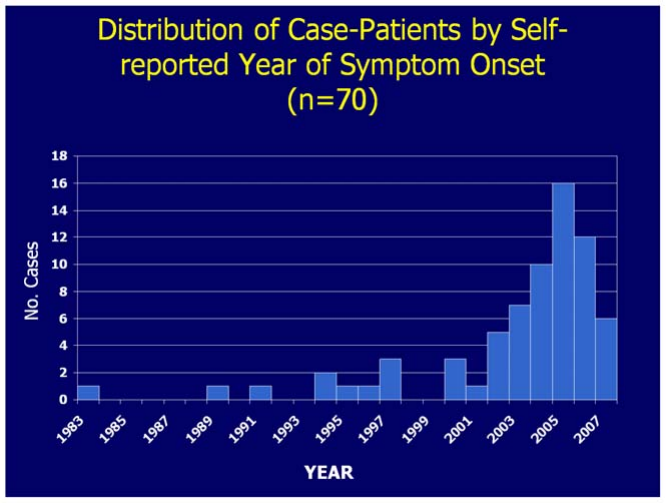
Distribution of Case-patients by Self-reported year of Onset, California.

The sociodemographic features of survey completers are shown in Table S1. The reported duration of symptoms ranged from 1.3 to 28.6 years (median: 3.7 years), with 69% participants reporting an illness duration of 2–5 years. The distribution of cases by reported year of illness onset is shown in Figure 3. Case-patients who reported “non-fibers” tended to report later illness onset (p = .057), but otherwise were similar to those who identified the material emerging from their skin as “fibers.”

#### Symptoms, Habits and Potential Exposures

Case-patients' symptoms are summarized in Table S2. There was no specific distribution to the skin symptoms, with 74% of case-patients stating that all areas of their body were affected. Half of case-patients described onset of skin symptoms as gradual. The reported sequence of skin symptom onset varied, with 57% of case-patients reporting disturbing skin sensations as the initial manifestation (followed either by skin lesions and/or solid material); 16% reported skin lesions as the initial manifestation; 10% the appearance of solid material as the initial manifestation; and 13% reported the triad of skin symptoms began simultaneously. Ten (14%) and seven (10%) case-patients, respectively, reported similar skin symptoms in a family member or friend.

No predominant temporal, diurnal or seasonal pattern to occurrence of skin symptoms or the emergence of material from the skin was reported, with 84% of respondents indicating that they had experienced skin symptoms “frequently” or “all the time” and 66% indicating that they noted fibers/solid material “anytime of the day.” Of the 14 (20%) who reported a seasonal occurrence of skin symptoms, 50% reported their symptoms as being worse during summer, 36% during spring, and 14% during winter.

Fibers and other material emerging from the skin were described as having a wide range of colors, with most (86%) being detected on skin areas with abnormal sensation; 73% reported having experienced the emergence of fibers or solid material from areas where there were no breaks in the skin.

Case-patients also reported a variety of non-skin symptoms involving multiple organ systems; non-skin symptoms reported by at least 50% of cases are shown (Table S2). Fatigue of ≥6 months duration and musculoskeletal complaints were among the most commonly reported, affecting 70% and 71% of case-patients, respectively.

When queried about habits and other potential exposures, some case-patients reported sharing personal items such as hairbrushes (18%), razors (13%), and towels (11%) or sharing a bed with another person (41%) or a pet (57%). Few case-patients reported using a hot tub (17%) or Jacuzzi (15%), residing in proximity to a land fill (3%), hazardous waste (3%) or industrial site (4%), or near live stock (3%) or orchards (1%) or travel in relation to symptom onset (20%). Most (78%) reported engaging, or having a household member who engaged, in hobbies or activities that involved the use of solvents (e.g., furniture stripper, paint thinner, turpentine, charcoal lighter fluid).

Case-patients reported using a wide range of topical and systemic over-the-counter, prescription and alternative therapies to alleviate their skin symptoms; no drug or treatment was consistently reported to be effective.

#### Health-related Quality of Life

Over 50% of case-patients rated their overall health status as fair or poor, a proportion significantly higher than reported among California residents or nationally (Table 2). Case-patients' PCS (mean = 36.63, SD  = 12.9) and MCS (mean = 35.45, SD = 12.89) scores also were significantly lower than expected national norms (mean = 50).

**Table 2:** Prevalence of Fair or Poor Self-rated Health Among Case-patients Completing Web-based Survey, Unexplained Dermopathy, California.

	% (95% Exact Confidence Interval)		
	Unexplained Dermopathy Study participants+N = 69	California*N = 26,097	US Population*N = 1,590,251
**Total**	53.6 (41.2–65.7)	17.5 (16.8–18.2)	16.3 (16.1–16.4)
**Sex**			
Male	30.0 (6.7 – 65.2)	16.6 (15.6–17.7)	15.3 (15.0–15.5)
Female	57.6 (44.1–70.4)	18.3 (17.5–19.2)	17.2 (17.0–17.4)
**Race/ethnicity**			
White, non-Hispanic	44.2 (30.5 – 58.7)	10.3 (9.7–10.9)	13.9 (13.7–14.0)
Black non-Hispanic	0.0 (0.0–45.9)**	22.2 (19.1–25.2)	20.4 (19.9–20.8)
Hispanic	60.0 (14.7–94.7)	27.9 (26.5–29.4)	26.5 (25.9–27.1)
Asian	0.0 (0.0–60.2)**	10.2 (8.3–12.0)	9.4 (8.6–10.1)

*Data are from the 1993–2007 Behavioral Risk Factor Surveillance System (BRFSS). All respondents to the BRFSS are non-institutionalized residents, 18 years old or older.

+ Excludes participants <18 yrs of age.

**One sided exact 95% confidence interval.

### Clinical Evaluations

Forty-one case-patients received clinical evaluation; they did not differ in sociodemographics from those who completed the survey but did not receive clinical evaluation (data not shown). On general clinical examination, six case-patients had muscle tenderness, one had cervical spine tenderness, and one had a positive Romberg. None had fever or lymphadenopathy.

At the time of clinical evaluation, 60% of case-patients rated the severity of their skin symptoms as five or greater (10 = most severe); 15 (37%) and 25 (61%), respectively, reported having fiber/material currently present or emerging from their skin within the previous 24 hours.

#### Type, Distribution and Severity of Skin Lesions

Clinical presentations varied substantially, including papules, scars, plaques, patches, macules, and one cyst. No case-patient had vesicles, bullae or burrows (suggestive of scabies). Many lesions were crusted, including some that were ulcerated or eroded. Some lesions and the surrounding area showed signs of inflammation (redness, warmth, tenderness).

**Figure 4:**
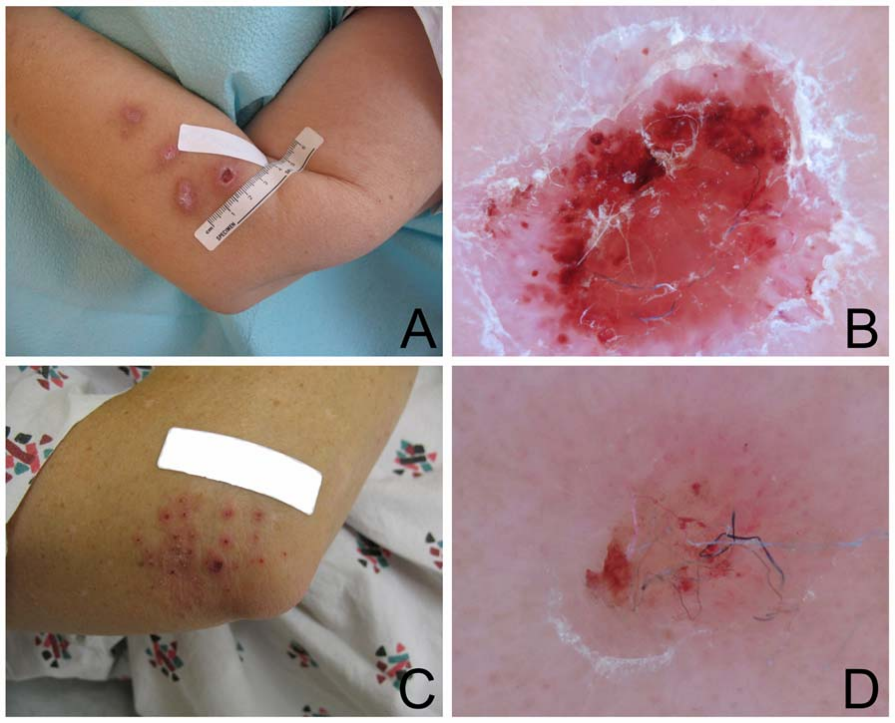
Representative skin lesions detected on clinical examination. **A.** Three erythematous scaly plaques with a fourth more proximal eroded and crusted plaque. **B.** Close-up of the eroded plaque in image 4A showing blue fibers. **C.** Excoriated erythematous papules suggestive of arthropod bites, dermatitis or possible excoriated folliculitis. **D.** Close-up of excoriated lesion in image 4C.

A median 17 lesions (range, 0–59) were documented per patient. The forearms (right, 83%; left, 71%), back (68%), chest (66%), face (66%) and lower legs (right, 63%; left, 66%) were the most commonly affected areas. Most arm lesions were on the posterior surface with sparing of the anterior surface. When back lesions were present, there was usually sparing of a dumbbell-shaped area in the center of the back. Clinically, the findings were most consistent with excoriations or chronic irritation, some with evidence of secondary infection. Representative skin findings are shown (Figure 4A–D).

#### Neurocognitive and Neuropsychiatric Assessments

Of 41 case-patients who underwent clinical evaluation, 36 (88%) completed the full battery of neuropsychological tests. Case-patients had estimated IQ scores ranging from 84 (low average) to 126 (high average), with a mean score of 109.9 (SD = 12.2).

On cognitive testing, 59% (23/39) case-patients demonstrated impairment in at least one domain; attention (18%) and memory (16%) were the most common areas of impairment. On the PAI, 63% (25/40) of case-patients had clinically significant elevations (T>70) in scores for one or more of the clinical domains, with somatic concerns the most frequent (63%), followed by depression (11%) (Table S3). Of the 24 case-patients with scores suggesting clinically significant somatic complaints, 14 (39%) had evidence of co-existing depression, 10 (37%) evidence of other co-existing neuropsychiatric conditions, and 12 (50%) had T scores >87, suggesting severe impairment arising from the somatic complaints. Four (24%) had evidence of clinically significant past or present drug or alcohol use.

### Laboratory Results

Few case-patients had abnormalities detected among the battery of blood tests; most were borderline abnormalities or abnormalities consistent with previously diagnosed conditions (e.g., diabetes, thyroid disease). Some case-patients had elevated markers suggestive of inflammation; five (12.5%) each had elevated RF or ESR, four (10%) elevated ANA, and three (7.5%) CRP.

Three (8%) case-patients had anti-HCV antibodies and five (12.5%, 2 borderline) anti-HBs antibodies. No case-patients had anti-HBc or HBsAg antibodies. One case-patient each had a positive or borderline EIA for *B. burgdorferi*, but none had a positive IgG WB. Three (8%) case-patients had positive serologies for *Toxocara* and three (8%) for *Strongyloides*.

At least one drug was detected in hair samples of 20/40 (50%) case-patients; these included amphetamines (3), barbiturates (1), benzodiazepines (8), cannabinoids (7), cocaine (2), opiates (8), and propoxyphene (1). All chest radiographs were interpreted as normal.

### Histopathologic and Microbiologic Features of Skin Biopsies

Of 41 case-patients who received clinical evaluation, 31 (75%) were deemed to have lesions amenable to biopsy or to have material that was present on the skin for collection. Biopsies (n = 62) were distributed across the entire body surface; 37 were from lesions, 22 from clinically normal skin and three were from undocumented sites.

**Figure 5:**
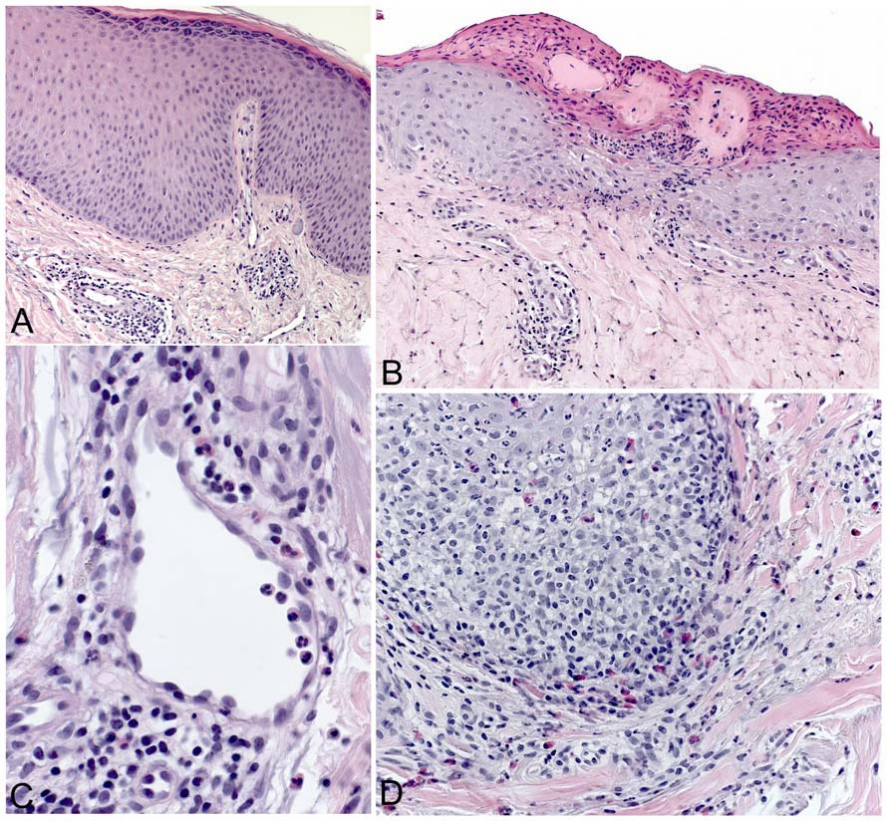
Representative histopathologic features of case-patient skin lesions. **A.** Epidermal hyperplasia with compact orthokeratosis and hypergranulosis and perivascular inflammatory infiltrates in the dermis consistent with lichen simplex chronicus. **B.** Focal erosion with superficial ulceration and scale-crust consistent with excoriation. **C.** Mixed perivascular inflammatory cell infiltrates comprised of lymphocytes, neutrophils and eosinophils, suggestive of arthropod bite or drug reaction. **D.** Suppurative folliculitis comprised of eosinophils and neutrophils. Hematoxylin and eosin stain, original magnifications ×25 (A, B), ×100 (C), and ×50 (D).

**Figure 6:**
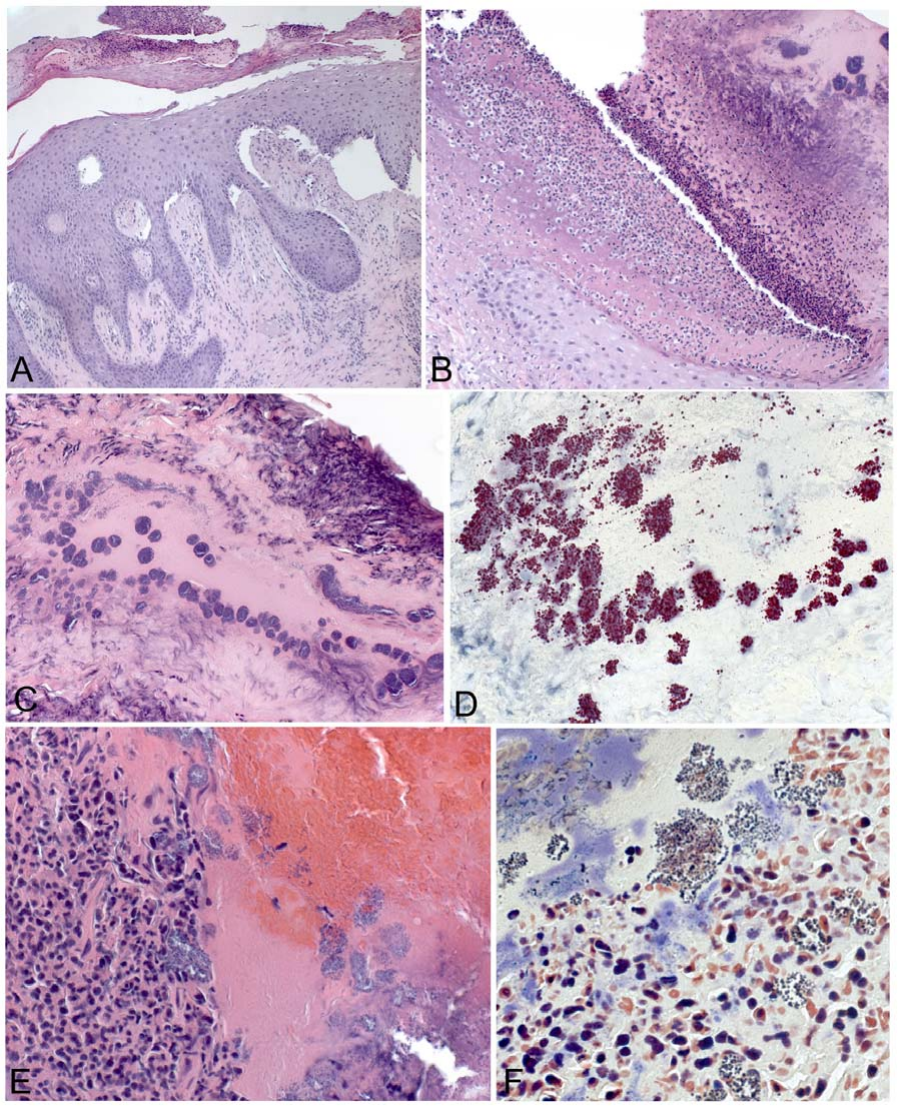
Superficial infectious processes identified in impetiginous skin lesions of case patients. **A.** Superficial and deep perivascular dermatitis with epidermal hyperplasia and prominent scale-crust. A heavy growth of *Stenotrophomonas maltophilia* was obtained in culture of this site. **B.** Ulcerated skin with purulent exudates and serum-crust containing numerous colonies of coccoid bacteria (**C**) that stain intensely by using an immunohistochemical technique for *Streptococcus pyogenes* **D** and **E**. Purulent serum-crust from an impetiginous lesion, with abundant colonies of gram-positive coccoid bacteria (**F**). A heavy growth of *Staphylococcus aureus* was obtained in culture of this site. Hematoxylin and eosin stain (A, B, C, F), immunoalkaline phosphatase with naphthol fast-red and hematoxylin counterstain (D), and Lillie-Twort stain (F). Original magnifications ×12.5 (A), ×25 (B), ×50 (C), ×100 (D, E), and ×158 (F).

Histopathologic features of the biopsied skin lesions were varied and representative findings are shown (Figures 5 and 6). Solar elastosis was the most common histopathologic abnormality, present in 19 (51%) biopsied lesions. Fifteen (40%) biopsied skin lesions showed histopathologic evidence of excoriation or chronic irritation (lichen simplex chronicus or prurigo nodularis); six (16%) others had features consistent with an arthropod bite or drug allergy.

Birefringent material was detected in 16 (43%) biopsied skin lesions. Most materials detected had the spectral characteristics of cellulose, compatible with cotton fibers. In all but two specimens, the birefringent material was located either in the superficial scale-crust, at the edge of or separate from the tissue, or on the biopsy surface and did not elicit a tissue reaction. Foreign-body-type giant cells were identified in two biopsies, one containing cellulose (most consistent with cotton fiber fragments), the other a silicon (Si)-containing material (likely silicates). Both of these biopsies had features suggestive of prior ulceration or trauma at the biopsy site.

By special stains, gram-positive bacteria or fungi were detected in 12 (11 participants) and eight (eight participants) specimens, respectively. For six of these specimens, IHC or PCR testing of the formalin-fixed tissues confirmed the bacteria as *Streptococcus pyogenes* (3), *Staphylococcus aureus* (2), or a *Streptococcus* sp. (1).

Culture swabs/specimens (n = 53) were obtained from open or purulent skin lesions of 28 case-patients. Organisms grew from the lesions of 15 case-patients; the histopathologic features of the culture-positive skin lesions were consistent with secondary infection (Figure 6). No skin lesions had detectable mycobacteria or parasites.

The 22 biopsies obtained from clinically normal sites were interpreted as histologically normal, except for solar elastosis (n = 5), sparse superficial perivascular inflammation (n = 5), chronic inflammation with rare eosinophils (n = 1), focal spongiosis, exocytosis and solar elastosis (n = 1) and a benign focal intradermal nevus. Five of these non-lesional biopsies contained cellulose fibers (resembling cotton), either adjacent to, or at the edge of, the biopsy; one had chronic perivascular inflammation and a birefringent material consistent with polyglycolic acid, a substance used in resorbable suture. None of the biopsies containing cellulose or polyglycolic acid had accompanying tissue reaction.

### Analysis of Fibers or Materials From Non-biopsy Skin Sites

**Figure 7:**
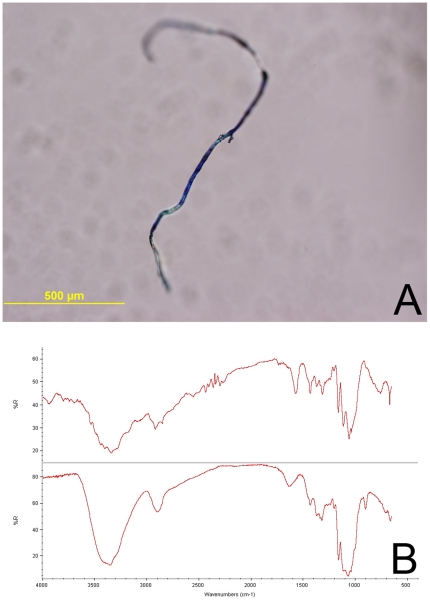
Spectral characteristics of fibers/materials. **A.** Photograph of formalin-fixed material with the IR spectral characteristics of cellulose consistent with a cotton fiber. **B.** Upper panel IR spectrum obtained from unidentified fiber, lower panel spectrum is a cellulose reference.

Twenty-three fiber or other material specimens were obtained from diverse intact skin sites in 12 case-patients. The materials were largely composed of protein (83%), likely superficial skin or cellulose consistent with cotton fibers (43%), some with evidence of dyes (Figures 7 A–B). Three samples contained other materials alone or in combination, including polyamide (probably nylon); cellulose nitrate containing bismuth (Bi) consistent with nail polish; and polyethylene (possible contaminant from specimen container lid).

## Discussion

In this study, we collected detailed epidemiologic, clinical and laboratory data to better characterize the features of an unexplained dermopathy often referred to as Morgellons. Among this study population, this unexplained dermopathy was rare, predominately affecting middle-aged, Caucasian, women. Over 75% of our cases reported onset of their symptoms during or after 2002, but the epidemiologic importance of this is unclear as it also corresponds to the time when Internet postings related to this condition began to surface. We did not identify clustering of illness within the geographic area served by KPNC and from which cases were drawn.

Case-patients had a wide range of skin lesions, suggesting that the condition cannot be explained by a single, well-described inflammatory, infectious, or neoplastic disorder. A substantial proportion (40%) of biopsied lesions had histopathologic features compatible with the sequelae of chronic rubbing or excoriation, without evidence of an underlying etiology. The most common histopathologic abnormality was solar elastosis, a degeneration of dermal connective tissue and increased amounts of elastic tissue due to prolonged sun exposure. However, this finding might be expected among a population residing in California and does not necessarily suggest a causal relationship. Histopathologic examination of skin areas with normal appearance were essentially normal, arguing against systemic or subclinical skin abnormalities. Among the differential diagnoses for the skin presentations detected are neurotic excoriations [16], atopic dermatitis, brachioradial pruritis [17], [18], and arthropod bites.

Previous reports of this condition have described the material emerging from the skin being like fibers, hairs or filaments [1], [19], but we found a more heterogeneous description of materials emerging from the skin, with many case-patients describing materials other than fibers including specks, dots, granules, or worms. We found no difference in the sociodemographic, clinical, or histopathologic characteristics of case-patients who did and did not report fibers. The fibers and materials collected from case-patients' skin were largely consistent with skin fragments or materials such as cotton and were either entrapped in purulent crust or scabs, suggesting the materials were from environmental sources (e.g., clothing) or possibly artifacts introduced at the time of specimen collection and processing.

We explored several possible etiologies and exposures. Our population had few clinical or laboratory signs of medical conditions that may be responsible for the symptoms, despite a wide range of accompanying multisystem complaints. We also did not find a pattern of clinical or epidemiologic abnormality that suggested any specific infectious etiology and, where data were available, the prevalence of specific parasitic infections in our population was no higher than that found in larger population-based studies [20]. We found evidence of drug use in 50% of participants. Formication can be a side affect drug use (prescription and illicit) and drug withdrawal, but the extent to which case-patients' drug use contributed to, or was being used as a treatment for, the condition was not determined. The high prevalence of drug use also may represent some case-patients' attempts to alleviate frustration or symptoms associated with the illness. Also, we found that over 75% of case-patients reported some exposure to solvents during hobbies. The prevalence of such exposures in the general population is unknown and we did not gather specific information regarding the types and duration of solvent exposures.

The prevalence of co-existing neuropsychiatric morbidity appeared to be high among our population based on measurements obtained by standardized screening instruments. Nearly 60% of case-patients had evidence of some cognitive impairment that could not be explained by deficits in IQ. Additionally, 63% of case-patients had clinically significant somatic complaints; nearly a third had somatic complaint scores that were elevated to levels rarely documented among other clinical populations but, when present, have been associated with chronic, multisystem complaints and incapacitating fatigue [5]. Lastly, we found functional impairment and disability (as measured by the SF-12) among the case-patients that exceeded that of the general population and comparable to that detected among persons who have serious medical illnesses and concurrent psychiatric disorders [21]–[23].

There are few studies of Morgellons in the medical literature with which to compare our study findings. In a report of 25 self-referred Morgellons patients, a minority (<1/3) had fibers detected at the time of examination and the most frequent dermatologic diagnosis was senile angiomas (72%); several patients had elevated cytokines (TNF-alpha, IL-6, IFN-gamma) [24]. We did not measure such markers in our study, but did find that a minority (15%) of case-patients had elevations in non-specific markers of inflammation, such as CRP and ESR. In another study of a convenience sample of Morgellons suffers from multiple states (46% from California), similar to our findings, those experiencing illness were predominantly Caucasian females and co-morbid conditions were common including a previous history of substance abuse (12%) and depression (29%) [25]. Neither study included biopsies or characterization of the materials obtained from patients' skin.

Our study had a number of limitations. This study was limited to KPNC enrollees who had current or recent symptoms (<3 months) thereby limiting our ability to describe the full clinical course of illness and to generalize the findings. However, our focus on persons with active or recent illness likely increased our ability to detect abnormalities and recover fibers or other materials. Our cross-sectional study design and lack of a comparison group did not allow us to determine the temporal relationship between symptoms and potential exposures or co-morbidities or to assess risk factors for illness. As there is no established definition or diagnostic test for this condition, our case definition was based on self-reported symptoms and hence subject to reporting biases and potential misclassification of cases. Some case-patients did not complete all phases of the study, but those who completed all phases of the study were demographically similar to those who did not. Lastly, we limited enrollment to persons at least 13 years of age.

Despite these limitations, our study provides a number of insights. The study was done among a well-defined and highly representative population of California, allowing generation of the first prevalence estimates of the condition and allowing us to look systematically for illness clustering. We extensively characterized the skin lesions afflicting case-patients, including systematic examination of intact and involved skin. We also performed detailed spectral and molecular analyses of fibers and other materials that have been reported as the condition's hallmark. Lastly, we assessed cognitive deficits, psychiatric co-morbidity and functional impairment among those affected.

To our knowledge, this represents the most comprehensive, and the first population-based, study of persons who have symptoms consistent with the unexplained dermopathy referred to as Morgellons. We were not able to conclude based on this study whether this unexplained dermopathy represents a new condition, as has been proposed by those who use the term Morgellons, or wider recognition of an existing condition such as delusional infestation, with which it shares a number of clinical and epidemiologic features [26]–[31]. We found little on biopsy that was treatable, suggesting that the diagnostic yield of skin biopsy, without other supporting clinical evidence, may be low. However, we did find among our study population co-existing conditions for which there are currently available therapies (drug use, somatization). These data should assist clinicians in tailoring their diagnostic and treatment approaches to patients who may be affected. In the absence of an established cause or treatment, patients with this unexplained dermopathy may benefit from receipt of standard therapies for co-existing medical conditions and/or those recommended for similar conditions such delusions infestation [31], [32].

## Supporting Information

Table S1 **Sociodemographic Characteristics of Case-patients Completing Web Survey, Unexplained Dermopathy, California (N = 70).**
(DOCX)

Table S2 **Skin and Non-Skin Symptoms Reported by Case-patients Completing Web Survey, Unexplained Dermopathy, California (N = 70).**
(DOCX)

Table S3 **Results of Personality Assessment Inventory Among Case-patients Completing Clinical Evaluation (N = 36).**
(DOCX)
