# Supplementary material for: Clinical, Epidemiologic, Histopathologic and Molecular Features of an Unexplained Dermopathy
Source: PLoS One. 2012 Jan 25;7(1):e29908. doi: 10.1371/journal.pone.0029908 (PMC3266263; doi:10.1371/journal.pone.0029908)
Supplement: Table S1 — Sociodemographic Characteristics of Case-patients Completing Web Survey, Unexplained Dermopathy, California (N = 70). (DOCX) [file pone.0029908.s001.docx]

Table S1. Sociodemographic Characteristics of Case-patients Completing Web Survey, Unexplained Dermopathy, California

(N=70)

| **Characteristic** | **Total**  **N=70** | **Fibers**  **N=51** | | **Non-Fibers**  **N=19** | **P-value** |
| --- | --- | --- | --- | --- | --- |
|  | **n (%)** | | | |  |
| **Age, yrs** |  |  |  | |  |
| Mean (SD) | 54.9 (13.9) | 55.9 (13.6) | 52.2 (14.7) | | .33 |
| Median (range) | 54.5 (17-93) | 55 (24-93) | 49 (17-93) | | .32 |
| **Age group** |  |  |  | |  |
| <18 yrs | 1 (1.4) | 0 | 1 (5.3) | | .34 |
| 18-44 yrs | 11 (15.7) | 8 (15.7) | 3 (15.8) | |  |
| 45-64 yrs | 46 (65.7) | 33 (64.7) | 13 (68.4) | |  |
| >=65 yrs | 12 (17.1) | 10 (19.6) | 2 (10.5) | |  |
| **Sex** |  |  |  | |  |
| Female | 59 (84.3) | 45 (88.2) | 14 (73.7) | | .14 |
| Male | 11 (15.7) | 6 (22.8) | 5 (26.3) | |  |
| **Race** |  |  |  | |  |
| White | 54 (77.1) | 39 (76.5) | 15 (79.0) | | .83 |
| African American/Black | 7 (10) | 4 (7.8) | 3 (15.8) | |  |
| Asian | 2 (2.9) | 2 (3.9) | 0 (0) | |  |
| Multi-racial | 3 (4.3) | 3 (5.9) | 0 (0) | |  |
| Other | 4 (5.7) | 3(5.8) | 1 (5.3) | |  |
| **Hispanic** |  |  |  | |  |
| Yes | 5 (7.1) | 4 (7.8) | 1 (5.3) | | .67 |
| No | 63 (90) | 45 (88.2) | 18 (94.7) | |  |
| Don’t know/not sure | 2 (2.9) | 2 (3.9) | 0 (0.0) | |  |
| **Marital Status** |  |  |  | |  |
| Married | 26 (37.1) | 18 (35.3) | 8 (42.1) | | .73 |
| Single, never married | 11 (15.7) | 7 (13.7) | 4 (21.0) | |  |
| Divorced | 22 (31.4) | 17 (33.3) | 5 (26.3) | |  |
| Separated | 2 (2.9) | 2 (3.9) | 0 | |  |
| Widowed | 6 (8.6) | 5 (9.8) | 1 (5.3) | |  |
| Domestic partner | 2 (2.9) | 2 (3.9) | 0 | |  |
| None of these | 1 (1.4) | 0 | 1 (5.3) | |  |
| **MRF registrant** |  |  |  | |  |
| Yes | 16 (22.9) | 12 (23.5) | 4 (21) | | .56 |
| No | 46 (65.7) | 32 (62.8) | 14 (73.7) | |  |
| Don’t know/not sure | 8 (11.4) | 7 (13.7) | 1 (5.3) | |  |
| **Education level** |  |  |  | |  |
| <High school | 2 (2.9) | 1 (2) | 1 (5.3) | | .61 |
| High school graduate/GED | 20 (28.6) | 13 (25.5) | 7 (36.8) | |  |
| Some college, no degree | 21 (30) | 15 (29.4) | 6 (31.6) | |  |
| Associate’s degree | 4 (5.7) | 4 (7.8) | 0 | |  |
| Bachelor’s degree | 10 (14.3) | 8 (15.7) | 2 (10.5) | |  |
| Some graduate/professional school, no degree | 5 (7.1) | 4 (7.8) | 1 (5.3) | |  |
| Graduate/professional degree | 7 (10) | 5 (9.8) | 2 (10.5) | |  |
| DK/NS | 1 (1.4) | 1 (2) | 0 | |  |
| **Employment history** |  |  |  | |  |
| Employed for wages | 17 (24.3) | 10 (19.6) | 7 (36.8) | | .32 |
| Self-employed | 6 (8.6) | 6 (11.8) | 0 | |  |
| Homemaker | 5 (7.1) | 4 (7.8) | 1 (5.3) | |  |
| Retired | 15 (21.4) | 13 (25.5) | 2 (10.5) | |  |
| Unable to work-disabled | 17 (24.3) | 12 (23.5) | 5 (26.3) | |  |
| Unemployed < 1 yr | 7 (10) | 4 (7.8) | 3 (15.8) | |  |
| Unemployed > 1 yr | 2 (2.9) | 2 (3.9) | 0 | |  |
| Never worked | 1 (1.4) | 0 (5.3) | 1 (5.3) | |  |
| **Occupation***** |  |  |  | |  |
| Healthcare worker | 9 (12.9) | 8 (15.7) | 1 (5.3) | | .43 |
| Homemaker | 9 (12.9) | 7 (13.7) | 2 (10.5) | | 1.00 |
| Office worker | 11 (15.7) | 9 (17.6) | 2 (10.5) | | .71 |
| Teacher | 4 (5.7) | 1 (2.0) | 3 (15.8) | | .06 |
| Other+ | 38 (54.3) | 27 (52.9) | 11 (57.9) | | .79 |
| **Duration of illness, yrs** |  |  |  | |  |
| Mean (SD) | 5.4 (4.8) | 5.9 (5.4) | 3.9 (2.3) | | .12 |
| Median (range) | 3.7 (1.3-28.6) | 3.8 (1.3-28.6) | 3.6 (1.9-11.7) | | .30 |
| **Duration of Illness** |  |  |  | |  |
| <2 yrs | 12 (17.1) | 8 (15.7) | 4 (21.0) | | .44 |
| 2-5 yrs | 37 (52.9) | 25 (49.0) | 12 (63.2) | |  |
| 6-10 yrs | 11 (15.7) | 9 (17.6) | 2 (10.5) | |  |
| >10 yrs | 10 (14.3) | 9 (17.6) | 1 (5.3) | |  |
| **Illness onset prior to 2002** |  |  |  | |  |
| Yes | 15 (21.4) | 14 (27.4) | 1 (5.3) | | .053 |
| No | 55 (78.6) | 37 (72.6) | 18 (94.7) | |  |
| **Primary residence type** |  |  |  | |  |
| Single family home | 44 (62.9) | 31 (60.8) | 13 (68.4) | | .09 |
| Townhouse/apartment/condo | 16 (22.9) | 10 (14.3) | 6 (31.6) | |  |
| Duplex/fourplex | 3 (4.3) | 3 (5.9) | 0 (0.0) | |  |
| Mobile home | 3 (4.3) | 3 (5.9) | 0 (0.0) | |  |
| Other/not sure | 4 (5.8.) | 4 (7.8) | 0 (0.0) | |  |
| **Additional persons in household** |  |  |  | |  |
| Mean (SD) | 1.62 (1.62) | 1.74 (1.66) | 1.32 (1.49) | | .33 |
| Median (Range) | 1.0 (0-7) | 1.00 (0-7) | 1.0/0-6 | | .30 |
| **Length of time at current residence** |  |  |  | |  |
| <1yrs | 8 (11.4) | 6 (11.8) | 2 (10.5) | | .36 |
| 1-5 yrs | 24 (34.3) | 15 (29.4) | 9 (47.4) | |  |
| >5 yrs | 38 (54.3) | 30 (58.8) | 8 (42.1) | |  |
| **Tobacco use ***** |  |  |  | |  |
| Ever smoked | 50 (71.4) | 35(68.6) | 15 (79.0) | | .40 |
| Current smoker | 27 (38.6) | 19 (37.3) | 8 (42.1) | | .71 |
| Daily smoker | 22 (31.4) | 16 (20.2) | 6 (31.6) | | .78 |
| **Substance use***** |  |  |  | |  |
| Ever | 45 (64.3) | 31 (60.8) | 14 (73.7) | | .41 |
| Past 30 days | 10 (14.3) | 8 (15.7) | 2 (10.5) | | .72 |
| **Pet owner** |  |  |  | |  |
| Yes | 54 (77.1) | 41 (80.4) | 13 (68.4) | | .29 |
| No | 16 (22.9) | 10 (19.6) | 6 (31.6) | |  |

MRF=Morgellons Research Foundation

SD=Standard deviation

+Contains 34 other occupations/occupational groups; none was larger than 1.

*** Categories not mutually exclusive
