# Supplementary material for: Clinical, Epidemiologic, Histopathologic and Molecular Features of an Unexplained Dermopathy
Source: PLoS One. 2012 Jan 25;7(1):e29908. doi: 10.1371/journal.pone.0029908 (PMC3266263; doi:10.1371/journal.pone.0029908)
Supplement: Table S2 — Skin and Non-Skin Symptoms Reported by Case-patients Completing Web Survey, Unexplained Dermopathy, California (N = 70). (DOCX) [file pone.0029908.s002.docx]

Table S2. Skin and Non-Skin Symptoms Reported by Case-patients Completing Web Survey, Unexplained Dermopathy, California

(N=70)

| **Symptom** | **%** |
| --- | --- |
| **SKIN** |  |
| **Emergence/excretion of material from skin*** | 100% |
| Fibers only | 3% |
| Fibers + non-fibers | 70% |
| Non-fibers only | 27% |
| **Disturbing skin sensations** | 99% |
| **Open sores/wounds/rash** | 90% |
| **NON-SKIN (reported by >50% cases)** |  |
| **General** |  |
| Fatigue | 70% |
| Persistent fatigue (all the time) | 74% |
| Fatigue not alleviated by rest (n=49) | 59% |
| Fatigue > 6 months duration | 70% |
| Unrefreshing sleep | 57% |
| Post-exertional fatigue | 49% |
| Sleep disturbance | 60% |
| Unplanned weight gain | 50% |
| **Eye, Ear, Nose and Throat** |  |
| Vision change | 60% |
| **Genitourinary** |  |
| Nocturia | 50% |
| **Gastrointestinal** |  |
| Bloating | 51% |
| **Musculoskeletal** |  |
| Muscle aches | 71% |
| Joint pain | 69% |
| Back pain | 67% |
| Difficulty moving joints | 57% |
| **Neurologic** |  |
| Difficulty finding words | 64% |
| Decreased memory | 59% |
| “Brain fog” | 59% |
| Numbness | 53% |
| Difficulty problem-solving | 50% |
| **Emotional or psychiatric** |  |
| Low energy | 71% |
| Anxiety | 59% |

*Required for case-definition
