# Supplementary material for: Clinical, Epidemiologic, Histopathologic and Molecular Features of an Unexplained Dermopathy
Source: PLoS One. 2012 Jan 25;7(1):e29908. doi: 10.1371/journal.pone.0029908 (PMC3266263; doi:10.1371/journal.pone.0029908)
Supplement: Table S3 — Results of Personality Assessment Inventory Among Case-patients Completing Clinical Evaluation (N = 36). (DOCX) [file pone.0029908.s003.docx]

Table S3. Results of Personality Assessment Inventory Among Case-patients Completing Clinical Evaluation

N=36

| **Clinical Scale** | **Mean T-score (SD)** | **Range** |  |
| --- | --- | --- | --- |
|  |  |  | **n (%) with**  **T-score >70** |
| **Somatic Complaints** | 77.3 (15.0) | 50-103 | 24 (64.9) |
| *Conversion* | 78.0 (17.5) | 51-108 | 23 (62.2) |
| *Somatization* | 69.9 (14.5) | 43-100 | 20 (54.1) |
| *Health Concerns* | 74.2 (13.0) | 50-94 | 23 (62.2) |
| **Anxiety** | 59.2 (11.0) | 38-89 | 6 (16.2) |
| *Cognitive* | 57.2 (10.8) | 38-85 | 5 (13.5) |
| *Affective* | 59.4 (11.2) | 39-89 | 4 (10.8) |
| *Physiological* | 58.5 (12.2) | 41-83 | 7 (18.9) |
| **Anxiety related Disorders** | 57.4 (11.8) | 31-93 | 4 (10.8) |
| *Obsessive-Compulsive* | 53.9 (13.4) | 28-81 | 7 (18.9) |
| *Phobias* | 53.9 (11.6) | 31-76 | 1 (2.7) |
| *Traumatic Stress* | 57.5 (12.7) | 41-99 | 4 (10.8) |
| **Depression** | 64.7 (13.6) | 43-94 | 14 (10.8) |
| *Cognitive* | 57.1 (12.9) | 40-87 | 5 (13.5) |
| *Affective* | 61.7 (14.0) | 39-94 | 9 (24.3) |
| *Physiological* | 67.4 (14.0) | 43-94 | 12 (32.4) |
| **Mania** | 52.8 (13.2) | 28-89 | 3 (7.1) |
| *Activity Level* | 51.1 (12.4) | 29-92 | 2 (5.4) |
| *Grandiosity* | 54.3 (12.5) | 31-81 | 4 (10.8) |
| *Irritability* | 51.1 (12.4) | 34-83 | 4 (10.8) |
| **Paranoia** | 54.3 (11.5) | 36-87 | 2 (5.4) |
| *Hypervigilance* | 55.8 (13.0) | 31-83 | 5 (13.5) |
| *Persecution* | 52.2 (12.5) | 39-104 | 1 (2.7) |
| *Resentment* | 52.8 (7.9) | 38-72 | 1 (2.7) |
| **Schizophrenia** | 57.0 (11.3) | 38-85 | 5 (13.5) |
| *Psychotic Experiences* | 49.4 (8.4) | 36-66 | 0 (0.0) |
| *Social Detachment* | 54.8 (12.7) | 36-79 | 6 (16.2) |
| *Thought Disorder* | 61.1 (11.2) | 37-87 | 7 (18.9) |
| **Borderline** | 55.0 (11.0) | 39-78 | 5 (13.5) |
| *Affective Instability* | 54.3 (11.9) | 39-85 | 5 (13.5) |
| *Identity Problems* | 53.4 (10.8) | 36-77 | 4 (10.8) |
| *Negative Relationships* | 57.7 (12.4) | 37-84 | 9 (24.3) |
| *Self-Harm* | 49.9 (10.5) | 37-76 | 1 (2.7) |
| **Antisocial Features** | 48.6 (7.4) | 37-74 | 1 (2.7) |
| *Antisocial Behaviors* | 48.0 (8.0) | 39-64 | 0 (0.0) |
| *Egocentricity* | 48.7 (7.0) | 39-75 | 1 (2.7) |
| *Stimulus-Seeking* | 48.4 (9.9) | 37-81 | 1 (2.7) |
| **Alcohol Problems** | 48.0 (7.7) | 41-72 | 1 (2.7) |
| **Drug Problems** | 55.1 (11.7) | 42-90 | 3 (7.1) |
